# Psychotic‐Like Reasoning Styles in Patients With Borderline Personality Disorder? An Experimental Investigation of the Jumping to Conclusions Bias

**DOI:** 10.1002/cpp.70051

**Published:** 2025-03-05

**Authors:** Jakob Scheunemann, Lisa Schilling, Christina Andreou, Steffen Moritz

**Affiliations:** ^1^ Department of Psychiatry and Psychotherapy University Medical Centre Hamburg‐Eppendorf Hamburg Germany; ^2^ Department of Psychiatry and Psychotherapy University of Lübeck Lübeck Germany

**Keywords:** beads task, cognitive distortions, decision‐making processes, probabilistic reasoning biases, psychosis, schizophrenia

## Abstract

**Introduction:**

Patients with borderline personality disorder (BPD) commonly display psychotic symptoms such as hallucinations or delusional/paranoid ideas. We used the fish task to investigate cognitive biases (jumping to conclusions and overcorrection) implicated in the aetiology of psychotic symptoms in patients with BPD.

**Methods:**

Participants received consecutive pieces of information to determine which of two lakes a fisherman was catching fish from. Outcome measures were draws to decision and frequency of premature decisions after just one and after not more than two fish (*jumping to conclusions*), probability estimate at the time of the decision (*decision threshold*) and adjustment of the probability estimate after receiving disconfirmatory information (*overcorrection*). With data aggregated from multiple studies, a total of 170 patients with BPD and 72 healthy controls (parallelized by age, gender and education) participated.

**Results:**

The two groups showed similar draws to decision and frequencies of premature decisions. The decision threshold was also comparable across the groups. However, the patients with BPD showed overcorrection.

**Conclusions:**

The experimental study found no evidence for a jumping to conclusions bias or a lower decision threshold in patients with BPD. The stronger adjustment of probability estimates (overcorrection) in patients with BPD is compatible with the unstable affect, self‐image and interpersonal relationships observed in patients with BPD.

**Summary**

- The fish task tested psychotic‐like cognitive biases in a transdiagnostic approach.
- Patients with borderline personality disorder show no jumping to conclusions bias.
- However, patients with borderline personality disorder show an overcorrection bias.
- Overcorrection aligns with symptoms such as instability in affect and self‐image.

## 1 Introduction

Borderline personality disorder (BPD) was initially conceptualised as a psychological condition at the interface of neurosis and psychosis or even a rather (mild) form of schizophrenia (Gunderson 1979; Liebowitz 1979). There is ongoing interest in the similarities and differences between patients with BPD compared to patients who meet diagnostic criteria for psychosis in relation to psychotic symptoms in patients (Zandersen et al. 2019). Almost half of patients with BPD report hallucinations (e.g., Kingdon et al. 2010; Niemantsverdriet et al. 2017; for reviews, see Belohradova Minarikova et al. 2022 and Merrett et al. 2016), which are indistinguishable from hallucinations in schizophrenia (Beatson et al. 2019; Tschoeke et al. 2014). Patients with BPD frequently display paranoid ideation (e.g., Kingdon et al. 2010 found that 29% of patients with BPD who did not meet criteria for schizophrenia reported paranoid delusions), schizotypal cognitions such as marked superstitiousness (e.g., George and Soloff 1986; Slotema et al. 2019) or ‘quasi‐psychotic’ thoughts, defined as delusions only present transiently or circumscribed in content (Zanarini et al. 1990).

This raises the question of whether the two disorders do have similar underlining etiological mechanisms as postulated decades ago. Regarding hallucinations, patients with BPD experiencing auditory verbal hallucinations share with patients with schizophrenia comparable subjective experiences (Hayward et al. 2022; Slotema et al. 2012), cognitive responses to the voices (Cavelti et al. 2019, 2020; Hepworth et al. 2013) and neural substrates (Strawson et al. 2022). Regarding decision‐making, direct comparisons in reward learning tasks indicate similar aberrant processes in information processing in both patient groups (Fineberg et al. 2018; Henco et al. 2020). Therefore, it appears valuable to further explore cognitive processes in individuals diagnosed with BPD that are characteristic of those with schizophrenia/psychosis, such as cognitive biases.

In schizophrenia/psychosis, research has elucidated several cognitive biases associated with delusions, such as overconfidence in errors and a bias against disconfirmatory evidence. Another prominent bias is the jumping to conclusions bias (often abbreviated as JTC; for reviews and meta‐analyses, see Dudley et al. 2016; McLean et al. 2017; So et al. 2016). The jumping to conclusions bias is defined as a hasty decision‐making style in which the individual makes decisions based on too little information (Evans et al. 2015). The bias is a central cognitive process in cognitive theories for the pathogenesis of delusions (e.g., Moritz, Göritz, et al. 2017; Ward and Garety 2019) and is a treatment target in multiple interventions such as metacognitive training (Moritz and Woodward 2007) or SlowMo (Garety et al. 2021).

Patients with psychosis demonstrate a jumping to conclusions bias in different experimental designs (e.g., Garety et al. 1991; Moritz, Pfuhl, et al. 2017; Serrano‐Guerrero et al. 2020; Veckenstedt et al. 2011). The classic original task is the beads task (Garety et al. 1991; Huq et al. 1988), with the fish task (Moritz et al. 2012) being a closely related variant of the same task. In the fish task, participants have to deduce which of two lakes a fisherman is catching fish from. Lake A contains more green than yellow fish and lake B contains more yellow than green fish (with ratios such as 80:20). After each fish is caught, the participant can decide which lake is being fished from or decide to see additional fish. In a study by Moritz and Woodward (2005), 42% of patients with psychosis decided after one catch/draw, compared to 7% of psychiatric controls and 0% of healthy controls. Based on a range of studies (for meta‐analyses, see McLean et al. 2017; So et al. 2016), the jumping to conclusions bias is robustly found in patients with psychosis. Interestingly, it is not just patients with full‐blown psychosis who show a jumping to conclusions bias; so do patients in remission (McLean et al. 2017), healthy participants with elevated levels of psychotic‐like experiences (Livet et al. 2020) and relatives of patients with psychosis (Henquet et al. 2020; Van Dael et al. 2006).

One reason to implicate a jumping to conclusions bias in patients with BPD is that patients with BPD show a range of cognitive biases, including dichotomous all‐or‐nothing thinking (e.g., Arntz and ten Haaf 2012; Puri et al. 2021) and biased interpretation of ambiguous information (Baer et al. 2012). Their decision‐making styles are disadvantageous, impulsive (Lawrence et al. 2010; Linhartová et al. 2021; Puri et al. 2021) and risky (Svaldi et al. 2012). Moreover, patients with BPD have unstable affect (D'Aurizio et al. 2023; Ebner‐Priemer et al. 2007; Pulcu et al. 2022) and unstable thoughts about themselves and others (Herzog et al. 2022; Kanske et al. 2016; Korn et al. 2016), which shows volatility and could influence decision‐making. Computational models of BPD also suggest that, in some instances, patients with BPD give new information too much weight in their decision‐making processes (Henco et al. 2020; Herzog et al. 2022). Taken together, patients with BPD might show a general bias in which decisions are based upon less information, resembling the jumping to conclusions bias found in patients with psychosis/delusions.

However, only a few studies have directly investigated the jumping to conclusions bias in BPD. On a self‐report measure conceptualised to capture cognitive biases associated with delusions (the Cognitive Biases Questionnaire for Psychosis; Peters et al. 2014), patients with BPD reported elevated scores in jumping to conclusions compared to healthy controls (Moritz et al. 2011). To the best of our knowledge, only one study has tested the beads task in patients with BPD (Catalan et al. 2015). At trend level, patients with BPD showed the jumping to conclusions bias more often than healthy controls. However, the sample size was small (patients with BPD only served as a psychiatric control group), and only one outcome of the beads task was reported. Importantly, researchers have already begun to address these biases therapeutically in patients with BPD, with some success (Schilling et al. 2018).

In line with the theoretical arguments and partial empirical evidence stated above, we expected patients with BPD to show a jumping to conclusions bias in the fish task, suggesting that patients with BPD—compared to healthy controls—make a premature decision more often (after the first or second draw), decide earlier (fewer draws to decision), have a lowered decision threshold (lower estimated probability at the time of the decision), and overcorrect their probability estimates more in line with disconfirmatory evidence. To test these hypotheses, we aggregated unpublished data from three previous studies by our research group. Moreover, we explored whether scores on the fish task correlated with psychometric measures such as self‐reported BPD symptom severity or specific symptoms (e.g., impulsiveness, dissociations) and basic neuropsychological measures (e.g., working memory, processing speed).

## 2 Material and Methods

### 2.1 Data Aggregation

For this study, we aggregated unpublished patient data from three studies (Andreou et al. 2015; Moritz et al. 2011; Schilling et al. 2015). Study 1 studied self‐reported cognitive biases in BPD and took place in 2010. Study 2 recruited patients from 2012 until 2014 and investigated social cognition. Study 3 investigated various cognitive processes in the period from 2014 to 2015 (Schilling et al. 2015). For the main outcomes we used two different fish tasks (described below). All participants completed fish task version 1, but not all completed fish task version 2. Across the studies, the study designs were almost identical (e.g., general procedures, diagnostics) and are described in detail below. However, the test batteries differed slightly (e.g., not all participants performed all tests). Regarding the main outcomes, the procedure for the fish task was identical across the studies. One participant participated in two of our studies; their data from the later study were removed from analysis. All data from the fish tasks by patients with BPD are unpublished; however, other outcomes have been reported previously. Fish task data from healthy controls were compiled from several studies, which have in part been published elsewhere (e.g., Moritz, Balzan, et al. 2016); results of the final sample of healthy controls, however, have never been published.

### 2.2 Participants

Across all three studies, all patients had received a BPD diagnosis according to the DSM‐IV before participation, additionally verified with the SCID‐II section on BPD (First and Gibbon 2004). A psychotic disorder according to the Mini International Neuropsychiatric Interview (M.I.N.I.; Sheehan et al. 1998) led to an exclusion. We only included healthy controls who did not meet the criteria for any current or lifetime psychiatric diagnosis according to the M.I.N.I. or self‐report. All participants had to be at least 18 years old and fluent in German.

### 2.3 Experimental Condition: The Fish Task

#### 2.3.1 Fish Task 1

The fish task (Woodward et al. 2009) is a variant of the beads task (Garety et al. 1991). In this task, participants deduce which of two lakes a fisherman is catching fish from. The two lakes have different proportions of orange and grey fish (lake A: 80% orange fish and 20% grey. lake B: 80% grey fish and 20% orange). The fisherman catches 10 fish in a fixed sequence: orange‐orange‐orange‐grey‐orange‐orange‐orange‐orange‐grey‐orange. After each new fish (also referred to as a *draw*), the participant estimates the probability from 0% to 100% that the fisherman is catching fish from lake A. In addition, the participant indicates whether the probability is sufficiently high to decide which lake the fisherman is fishing from (simulated decision). Independent of the participant's answer the experiment proceeds, meaning that all participants see the full sequence of 10 fish.

An instructor was present at all times; they read out the standardised instructions, asked for any questions about the task, answered open questions and made it clear that the fisherman would not change lakes. If the participant changed their decision on the lake, the instructor reminded the participant that the lake would not change during the experiment. Participants saw the task illustrated on a computer but gave their responses out load verbally, which the instructor then wrote down. At all times, to keep memory load at a minimum, participants saw the current and all previously caught fish as well as the overall proportions of the fish for the two lakes.

#### 2.3.2 Fish Task 2

The procedure of fish task 2 was identical to fish task 1 except that after each new fish (*draw*) the participant did not give a probability rating but indicated only whether they wanted to decide on a lake. If the participant made a decision, the experiment was terminated. The sequence was identical to fish task 1 but used different colours (red and green fish instead of grey and orange).

#### 2.3.3 Scoring of the Fish Tasks

The main outcome was draws to decision. Draws to decision is the number of fish the participant had seen before making their first decision on a lake. If the participant did not decide after the tenth fish, the score was set to 11. Thus, the possible score ranged from 1 to 11. As done previously (Van Dael et al. 2006), we calculated the frequency of decisions after the first draw (draws to decision ≤ 1) or after no more than two draws (draws to decision ≤ 2). Furthermore, we calculated the decision threshold, defined as the probability rating the participant gave for Lake A when they first decided that the fisherman was fishing from Lake A. Finally, the fourth and the ninth fish presented disconfirmatory evidence for lake A, which allowed us to calculate an overcorrection measure, as described by Garety et al. (1991). We subtracted the probability estimate after Fish 3 from the probability estimate after Fish 4 and averaged it with the same subtraction from the probability estimate after Fish 9 compared to the estimate after Fish 8.

#### 2.3.4 Post‐Questionnaire About the Fish Task

After the task, participants filled out a paper‐and‐pencil questionnaire with the following questions: (1) Did you understand the task (responses: (a) yes, (b) no, (c) unsure); (2) What do you think was expected from you in the task (multiple responses possible: (a) determination, making a decision after as few fish as possible, (b) caution, meaning to wait for many fish before making a decision, (c) accuracy, meaning to make the right decision any way possible, (d) speed, meaning to give a fast response, (e) I cannot say).

### 2.4 Psychopathological and Neuropsychological Measures

We report nine outcomes scores from seven self‐report measures as well as 10 outcome scores from seven neuropsychological tests. As not all measures and tests were included in the three studies, the results for each measure are reported for subsamples with varying sample sizes. We describe all measures in depth in the supplementary materials (S1). The psychopathological measures capture BPD symptom severity (Borderline Symptom List, BSL‐23; Bohus et al. 2009), depression symptoms (Beck Depression Inventory‐II, BDI‐II; Beck et al. 1996), dissociative symptoms (Dissociative Experiences Scale‐Taxon; Waller et al. 1996), and impulsivity (Barratt Impulsiveness Scale 11th revision; Patton et al. 1995). As cognitive measures, we captured BPD specific cognitions and feelings (11‐item short version of the Questionnaire of Thoughts and Feelings; Renneberg and Seehausen 2010), cognitive insight (Beck Cognitive Insight Scale; Beck et al. 2004), and cognitive biases related to psychosis (Cognitive Biases Questionnaire for Psychosis; Peters et al. 2014). The neuropsychological measures were vocabulary tests as measures for premorbid general intelligence (Lehrl et al. 1995; Metzler and Schmidt 1992), tests on memory (Rivermead Behavioural Memory Test; Wilson et al. 1989), processing speed and executive functioning (Trail making Tests A and B; Reitan and Wolfson 1995), and attention and working memory (digits forward and digits backward of the Wechsler Adult Intelligence Scale‐Revised; Wechsler 1981). We also conducted the Mini International Neuropsychiatric Interview (M.I.N.I.; Sheehan et al. 1998) to verify exclusion criteria and to report comorbidities.

### 2.5 Analysis

We calculated group differences for the five outcome scores of the fish task with Welch's *t*‐tests. Further, we report the effect size measure using Cohen's *d* (|*d*| ≈ 0.2 small, |*d*| ≈ 0.5 medium, |*d*| ≈ 0.8 large) and the corresponding 95% confidence intervals. To assess associations between the psychopathological/neuropsychological measures and fish task outcome scores, we calculated Pearson correlations for metric outcome scores (draws to decision, decision threshold and overcorrection) as well as odds ratios for binary outcome scores (decision after one draw and decision after one or two draw(s)).

#### 2.5.1 Matching

We had fish task data from 170 patients with BPD and 98 healthy controls. We matched healthy controls to the patients with BPD based on the variables of age, gender and education (A level vs. not A level). We used cardinality matching of the MatchIt R package (Randolph et al. 2014), performed by the solver Gurobi (Gurobi Optimization, LLC 2023). We set the maximum mean differences between the matched samples to 0.2 standard deviations for all covariates. The resulting group sizes were 170 patients with BPD and 72 healthy controls. No between‐group differences occurred for age (BPD: *M* = 27.9 [*SD* = 7.6], HC: *M* = 29.4 [*SD* = 10.6], *t*(103.63) = 1.03, *p* = 0.306), gender (BPD: 87.6% female, HC: 81.9% female; *Χ* ^2^(1) = 0.93, *p* = 0.335) or education (BPD: 44.1% A level, HC: 52.8% A level; *Χ* ^2^(1) = 1.20, *p* = 0.274). A sensitivity power analysis revealed that the given sample sizes (*n* = 170 and *n* = 72) could detect effect sizes of *d* ≥ 0.35 with a power of *β* = 0.80 and *α* = 0.05 (as calculated using G*Power 3.1.9.7 [Faul et al. 2007]).

## 3 Results

Table 2 shows means and standard deviations of psychopathological and neuropsychological scores. Symptom severity in the patient group was high. For example, patients self‐reported borderline symptom load in the BSL‐23 comparable to the validation sample (46.8; Bohus et al. 2009, who reported a mean of 47.2). Within the patient group, the majority (55.1%) had scores above 29 on the BDI‐II, indicating severe depression and only 3.8% had scores below 14, indicating no current depression. According to the M.I.N.I. (subsample of *n* = 159 [94% of total BPD sample]), 96.9% reported at least one comorbidity (*M* = 2.5, *SD* = 1.2), most often current (75.5%) or lifetime (92.5%) depression. For details on the frequencies of comorbidities, see Table S1 (supplementary materials). In total, 138 of 170 (81.2%) patients were currently taking psychopharmacological medication.

### 3.1 Main Analyses: Group Differences in the Fish Task

We compared patients with BPD to healthy controls on their performance on both fish tasks (see Table 1). Patients with BPD did not show a jumping to conclusions bias in any of the calculated scores. First, patients with BPD and healthy controls both decided for a lake after around four draws/fish, with almost identical scores at a negligible effect size. Second, patients with BPD and healthy controls had similar ratios of premature decision after the first draw/fish (14% vs. 18%) or not later than the second draw/fish (31% vs. 38%). Results were replicated in fish task 2 in smaller subsamples, with slightly more careful behaviour in both groups (more draws, fewer premature decisions).

**TABLE 1:** Group differences and effect sizes on the outcomes of the two fish tasks.

	Patients with BPD	Healthy controls	Statistics	*p*	Effect size	95% CI Effect size
**Fish Task 1 (Threshold version)**						
Sample size	*n* = 170	*n* = 72				
Draws to decision	4.1 (*SD* = 2.4)	4.0 (*SD* = 2.7)	*t*(120.98) = 0.09	0.925	*d* = −0.02	[−0.26; 0.30]
Decision after 1 draw (yes/no)	24/146 (14.1% yes)	13/59 (18.1% yes)	*Χ* ^2^ (1) = 0.34	0.560	*φ* = 0.00	[0.00; 0.15]
Decision after 1 or 2 draws (yes/no)	52/118 (30.6% yes)	27/45 (37.5% yes)	*X* ^2^ (1) = 0.81	0.369	*φ* = 0.00	[0.00; 0.17]
Decision threshold^a^	81.2 (*SD* = 21.3)	82.9 (*SD* = 15.8)	*t*(167.84) = 0.67	0.502	*d* = −0.09	[−0.37; 0.20]
Overcorrection	3.5 (*SD* = 12.0)	1.1 (*SD* = 6.1)	*t*(233.06) = 2.04	0.042	*d* = 0.22	[−0.05; 0.50]
**Fish Task 2 (No threshold version)**						
Sample size	*n* = 142	*n* = 57				
Draws to decision	4.5 (*SD* = 2.2)	4.3 (*SD* = 2.2)	*t*(104.65) = 0.40	0.691	*d* = 0.06	[−0.25; 0.37]
Decision after 1 draw (yes/no)	8/134 (5.6% yes)	2/55 (3.5% yes)	*X* ^2^ (1) = 0.68	0.794	*φ* = 0.00	[0.00; 0.13]
Decision after 1 or 2 draws (yes/no)	29/113 (20.4% yes)	11/46 (19.3% yes)	*X* ^2^ (1) = 0.00	1	*φ* = 0.00	[0.00; 0.00]

*Note:*

^a^ Sample sizes *n* = 164 for BPD and *n* = 68 for healthy controls; BPD = borderline personality disorder; CI = confidence interval.

Fish task 1 allowed us to calculate a decision threshold and a measure for overcorrection. Again, no significant group differences emerged for the decision threshold measure. Moreover, the probability ratings did not differ between patients with BPD and healthy controls after any of the 10 probability ratings (*p* ≥ 0.078, *d* ≤ 0.22). However, patients with BPD corrected their probability estimates significantly more in response to disconfirmatory evidence (the rarer grey fish shown at Draws 4 and 9), with a small effect size.

### 3.2 Associations With Psychopathology and Neuropsychology

Within the group of patients with BPD, we correlated several clinical and neuropsychological measures with the fish task scores. Table 2 reports all 95 computed correlations and odds ratios. Even when dismissing alpha‐error accumulation for multiple testing, the significant correlations were only between processing speed/executive functioning (Trail Making Test A, Trail Making Test B) and draws to conclusion and the only significant odds ratio was between impulsivity and a premature decision after two or less draws, all with a small effect size. All of the correlations were within |*r*| < 0.30 and all odds ratios were *O* *R* < 1.2 (Chen et al. 2010 found an *OR* < 1.5 to be equivalent to Cohen's *d* < 0.2), indicating a lack of meaningful association between psychopathological, cognitive or neuropsychological measures and fish task performance in patients BPD.

**TABLE 2:** Means, standard deviations, correlations and odds ratios between psychopathological and neuropsychological measures and performance on fish task 1 in patients with BPD.

				Draws to decision	Decision after 1 draw	Decision after 1 or 2 draw(s)	Decision threshold	Overcorrection
	*n* ^a^	Mean	*SD*	*r*	*OR*	*OR*	*r*	*r*
**Clinical measures**								
Borderline symptom list	160	46.8	18.9	−0.01	1.01	1	0.08	−0.12
Borderline symptom list–behaviour scale	158	4.5	4.3	−0.07	1.05	1.02	−0.10	−0.05
Beck depression inventory‐II	156	29.9	10.3	0.01	0.99	1.01	0.01	−0.05
Dissociative experiences scale–taxon	151	155.8	128.3	−0.01	1.00	1.00	0.08	−0.07
Barratt impulsiveness scale	46	73.8	9.7	−0.29	1.16	**1.14** *	−.0.17	0.11
Questionnaire of thoughts and feelings	64	47.2	10.3	0.06	1.02	1.00	−0.19	0.02
Beck cognitive insight scale–self‐reflectiveness	71	13.0	3.4	0.06	1.04	0.93	−0.18	0.01
Beck Cognitive Insight Scale–Self‐Certainty	71	7.1	3.1	−0.09	1.05	1.11	−0.08	0.09
Cognitive biases questionnaire for psychosis	28	47.2	8.1	−0.28	1.13	1.02	−0.19	−0.06
**Neuropsychological measures**								
Vocabulary test (WST)	62	29.2	4.8	−0.07	0.92	1.03	−0.15	0.08
Vocabulary test (MWT‐B)	109	27.3	4.6	0.00	1.05	0.98	−0.06	0.02
Rivermead behavioural memory test—immediate recall	142	7.7	3.2	−0.04	0.94	0.94	−0.13	0.09
Rivermead behavioural memory test—delayed recall	141	6.9	7.4	0.19	0.97	0.92	−0.08	0.00
Trail making test A in sec	168	24.2	8.1	**−0.19** *	1.03	1.03	0.01	0.02
Trail making test A percentile	168	53.0	27.9	**0.** **16** *	0.99	0.99	0.00	0.01
Trail making test B in sec	169	58.4	22.7	−0.10	1.01	1.00	0.02	0.08
Trail making test B percentile	169	45.3	33.8	**0.** **16** *	0.99	1.00	−0.04	−0.04
Digits forward (WAIS‐R)	81	10.6	2.7	−0.12	1.01	1.12	0.07	−0.03
Digits backward (WAIS‐R)	81	7.0	2.4	−0.12	0.92	1.01	0.13	−0.19

*Note:*

* *p* < 0.05; WST = Wortschatztest; MWT‐B = Mehrfachwahl‐Wortschatz‐Intelligenztest, WAIS‐R = Wechsler Adult Intelligence Scale‐Revised.

^a^ For decision threshold, sample sizes were ≤ 6 participants fewer, from top to bottom: 154, 152, 150, 145, 43, 61, 68, 68, 28, 59, 106, 136, 135, 162, 162, 163, 163, 78, 78.

### 3.3 Correlations Among Fish Task Scores

Small positive correlations emerged between the decision threshold and draws to decision in both groups, reaching significance for patients with BPD (*r*(162) = 0.16, *p* = 0.039) but not for healthy controls (*r*(66) = 0.17, *p* = 0.175). Overcorrection was not significantly associated with draws to decision in any group (BPD: *r*(168) = −0.14, *p* = 0.078; healthy controls: *r*(70) = 0.09, *p* = 0.461). The association between the decision threshold and overcorrection revealed a complex picture. In patients with BPD, a lower decision threshold correlated largely with more overcorrection (*r*(162) = −0.50, *p* < 0.001), whereas for healthy controls the correlation was in the opposite direction (*r*(66) = 0.20, *p* = 0.105). The difference in correlation is significant according to the corresponding Fischer's test (*r*_difference_ = 0.70, *z* = 5.13, *p* < 0.001), as calculated using the R package *cocor* (Diedenhofen and Musch 2015).

### 3.4 Subjective Understanding of the Fish Task

A subsample of 140 patients with BPD and 57 healthy controls responded to a questionnaire on their subjective understanding of the fish task. No significant group differences between patients and healthy controls emerged for subjective comprehension of the task: 131 of 140 (93.6%) of patients with BPD versus 56 of 58 (96.6%) of healthy controls said they understood the task; the remaining participants were mostly unsure (seven patients [5.0%] and two healthy controls [3.4%]), with only two patients (1.4%) and no healthy control responding that they had not understood the task (*Χ* ^2^(2) = 1.08, *p* = 0.582). Regarding their assumption concerning what was expected of them for the task (participants could give multiple answers), responses were similar between patients with BPD and healthy controls, with no significant group differences: Most participants thought the purpose of the task was to test determination (BPD: 57.9%, healthy controls: 65.6%, *Χ* ^2^(1) = 0.71, *p* = 0.400). The other options received less agreement: accuracy (BPD: 39.3%, healthy controls: 32.8%%, *Χ* ^2^(1) = 0.49, *p* = 0.482), caution (BPD: 27.9%, healthy controls: 41.4%, *Χ* ^2^(1) = 2.86, *p* = 0.091) and speed (BPD: 16.7%, healthy controls: 23.4%, *Χ* ^2^(1) = 0.37, *p* = 0.542). Only a minority of participants responded that they could not say what the purpose of the task was (BPD: 12.9%, healthy controls: 3.4%, *Χ* ^2^(1) = 3.03, *p* = 0.082).

## 4 Discussion

This study explored the jumping to conclusions bias in patients with BPD. We compared performance between patients with BPD and healthy controls on the fish task, a variant of the beads task (Garety et al. 1991) frequently used in schizophrenia research. In the fish task (Woodward et al. 2009), participants receive successive information (the colour of fish caught) and make judgements based on this information (which of the two lakes the fish are being caught from). The fish task allowed us to investigate whether patients with BPD show the following cognitive biases that have been linked to the aetiology of delusions and psychosis: jumping to conclusions (i.e., premature decision‐making based on too little information), a lowered decision threshold (i.e., making a decision at a lower probability estimate) and overcorrection (i.e., overly strong adjustment of the probability estimate).

### 4.1 Jumping to Conclusions

Contrary to our hypothesis, patients with BPD had the same number of draws to decision as healthy controls. Furthermore, they did not decide more often prematurely (after one or after no more than two draws). Thus, patients with BPD did not show a jumping to conclusions bias on the experimental fish task. These results are in line with Scheunemann et al. (2023), who did not find that patients with BPD sought less advice in an estimation task (as a proxy for information seeking/jumping to conclusions). However, these experimental studies are at odds with research in which patients with BPD indicate a jumping to conclusions bias in self‐report (Moritz et al. 2011; Puri et al. 2018). It is important to note that experimentally tested JTC does not correlate highly with self‐reported JTC in patients with schizophrenia (Gaweda et al. 2017; Moritz, Balzan, et al. 2016; Peters et al. 2014). More research and possibly new experimental designs are necessary to reproduce patients' self‐perceived hasty decision‐making style and clinical observations.

### 4.2 Decision Threshold

Patients with BPD also did not show a lowered decision threshold, contrary to our hypothesis. In fact, patients with BPD and healthy controls estimated the probability similarly at all points of the experiment, including the time of the decision. To conclude, patients did not judge the objective probability differently than healthy controls, and they had a similar threshold for their decision. Thus, their behaviour differed from the behaviour observed in patients with schizophrenia, who tend (on some tasks) to give similar probability ratings to controls but decide at a lower probability estimate/decision threshold (Moritz et al. 2006; Moritz, Scheu, et al. 2016). This lowered decision threshold has been argued to explain the jumping to conclusions bias found in the fish task (Moritz et al. 2012) and is an integral part of the two‐stage theory for the development of delusions (Moritz, Pfuhl, et al. 2017). The absence of both the jumping to conclusions bias and the lower decision threshold found in our BPD sample speaks for different cognitive etiological mechanisms in psychosis and BPD.

### 4.3 Overcorrection

As expected, patients corrected their probability estimates more than healthy controls in response to disconfirmatory information. As the fisherman catches mostly orange fish, participants should lower their probability rating that the fisherman is catching from the lake with predominantly orange fish, whenever the fisherman catches a grey fish. In line with the hypothesis, patients with BPD lowered their ratings significantly more than healthy controls. Thus, patients corrected their probability ratings too strongly based on the new information; i.e., patients with BPD showed overcorrection, just as patients with psychosis show overcorrection on the same task (Balzan et al. 2012; Colbert and Peters 2002; Klein and Pinkham 2018).

In psychosis research, several studies have investigated the fish task (and its variants) and suggested that overcorrection can be explained by an overly high weighting of the most recent information, as in, for example, the hypersalience of evidence‐matching hypothesis model (Speechley et al. 2010), unstable‐attractor network (Adams et al. 2018; Hauke et al. 2022), and circular inference (Jardri et al. 2017). As mentioned above, computational models posit similar aberrant mechanisms in patients with BPD and patients with schizophrenia (Fineberg et al. 2018; Henco et al. 2020). For BPD, a recently postulated predictive processing model explains the instability regarding affect, self‐image and interpersonal relationships observed in patients with BPD by their over‐reliance on sensory evidence due to weak predictive priors (Herzog et al. 2022). The theory outlines how patients with BPD might develop this mechanism as a consequence of repeated childhood maltreatment. This study adds some experimental evidence for this theory. It seems worthwhile to investigate the mechanisms underlying overcorrection. Notable in this regard is the finding that the correlation between decision threshold and overcorrection was negative in the patient group but positive in the healthy controls.

### 4.4 Robustness of Findings

We correlated the outcome measures of the fish task with a wide range of clinical and neuropsychological measures. All correlations were weak and insignificant (when neglecting alpha‐error accumulation for multiple testing, a few correlations of very small effect size reached significance). Thus, performance on the fish task does not seem to be influenced by the severity of borderline symptomatology, depression severity, dissociative symptoms, impulsivity, BPD specific cognitions, cognitive insight or subjective cognitive biases related to psychosis. Moreover, performance on the fish task was also not predicted by measures of neuropsychological functioning such as word fluency (as a measure for premorbid intelligence), memory, executive functioning, attention or working memory. Only processing speed correlated at a low level with one outcome measure. Thus, it is unlikely that a BPD sample with a different symptom pattern (e.g., less or more BPD symptomatology or a different phenotype) would have yielded different results or that the findings might be confounded by neuropsychological deficits.

### 4.5 Integration of Results With Previous Findings and Outlook

Evidence from this study suggests that patients with BPD do not show the jumping to conclusions bias, which is considered relevant for the pathogenesis of psychotic symptoms. While meta‐analyses support the presence of a jumping to conclusions bias in psychosis, some large‐scale studies have failed to replicate the bias in patients with psychosis (Pytlik et al. 2020; Tripoli et al. 2019). Moreover, new experimental designs have cast additional doubt about this bias (Moritz et al. 2020). In clinical research across disorders, the beads task has also failed to discriminate healthy controls from clinical populations, for example, those with attention deficit hyperactivity disorder (Lunt et al. 2012), anxiety disorder (Jacoby et al. 2014), anorexia nervosa (McKenna et al. 2014) and bipolar disorder (Can et al. 2019). Future studies in both psychosis and BPD research, as well as in research on various other disorders, should work to refine the experimental methodology to measure the thinking styles that align with the clinical observations.

Our approach to applying experimental designs from psychosis research to investigate etiological mechanisms in BPD seems worth pursuing, especially as similar etiological mechanisms between BPD and psychotic disorders have been identified in the past (e.g., Fineberg et al. 2018; Henco et al. 2020). As patients with BPD show divergences, especially in interpersonal settings (Lazarus et al. 2014) or in emotional contexts (Baer et al. 2012; Kaiser et al. 2017), it is possible that a hasty decision‐making style would be found in relation to emotional stimuli or within social interactions but not in the BPD‐unrelated probabilistic task used in this study. To measure jumping to conclusions using emotional stimuli or in a social setting, new experimental designs are needed (for approaches, see Scheunemann et al. 2020; Warman and Martin 2006; Westermann et al. 2012).

### 4.6 Strengths and Limitations

One strength of this study is the large sample size of well‐diagnosed patients with BPD, with high symptom burden. Furthermore, we calculated multiple outcomes of two versions of the task and correlated task performance with a range of measures.

The study has some limitations, including the lack of a psychiatric control group (e.g., patients with depression but without BPD). It is important to mention that we aggregated the data for patients with BPD and healthy controls from multiple studies conducted in our lab. Thus, while the main outcome measure was consistent across studies, some heterogeneity may still have arisen due to differences in study procedures, recruitment strategies or assessment conditions. Future studies should investigate whether the subsample of patients with BPD who actually report psychotic symptoms show the jumping to conclusions bias.

## 5 Conclusion

In this study, we experimentally investigated whether patients with BPD show cognitive biases that are associated with the pathogenesis of psychosis (i.e., psychotic‐like cognitive biases). To do this, we used the well‐established fish task, a variant of the beads task. Patients with BPD showed no jumping to conclusions bias and no divergent decision threshold, but they did show overcorrection. Thus, we did not replicate experimentally the clinical observation that patients with BPD make hasty decisions. However, the patients' strong adjustments in their probability estimates in response to new and disconfirmatory information (overcorrection bias) correspond with the rapidly alternating beliefs many patients with BPD often show. Future studies may build upon these findings to investigate reasoning biases as potential transdiagnostic psychological mechanisms across disorders.

## Supporting information


**Data S1.** Psychopathological and neuropsychological measures.

**Table S1.** Frequency of comorbidities based on the Mini International Neuropsychiatric Interview (M.I.N.I.) in a sub sample of *n* = 159 patients (93.5% of total BPD sample).

## Data Availability

Data will be made available upon request.

## References

[cpp70051-bib-0001] Adams, R. A. , G. Napier , J. P. Roiser , C. Mathys , and J. Gilleen . 2018. “Attractor‐Like Dynamics in Belief Updating in Schizophrenia.” Journal of Neuroscience 38, no. 44: 9471–9485. 10.1523/JNEUROSCI.3163-17.2018. 30185463 PMC6705994

[cpp70051-bib-0114] Andreou, C. , L. Kelm , J. Bierbrodt , et al. 2015. “Factors Contributing to Social Cognition Impairment in Borderline Personality Disorder and Schizophrenia.” Psychiatry Research 229, no. 3: 872–879. 10.1016/j.psychres.2015.07.057. 26257087

[cpp70051-bib-0002] Arntz, A. , and J. ten Haaf . 2012. “Social Cognition in Borderline Personality Disorder: Evidence for Dichotomous Thinking but No Evidence for Less Complex Attributions.” Behaviour Research and Therapy 50, no. 11: 707–718. 10.1016/j.brat.2012.07.002. 22982582

[cpp70051-bib-0003] Baer, R. A. , J. R. Peters , T. A. Eisenlohr‐Moul , P. J. Geiger , and S. E. Sauer . 2012. “Emotion‐Related Cognitive Processes in Borderline Personality Disorder: A Review of the Empirical Literature.” Clinical Psychology Review 32, no. 5: 359–369. 10.1016/j.cpr.2012.03.002. 22561966

[cpp70051-bib-0004] Balzan, R. P. , P. H. Delfabbro , C. A. Galletly , and T. S. Woodward . 2012. “Over‐Adjustment or Miscomprehension? A Re‐Examination of the Jumping to Conclusions Bias.” Australian and New Zealand Journal of Psychiatry 46, no. 6: 532–540. 10.1177/0004867411435291. 22679205

[cpp70051-bib-0006] Beatson, J. A. , J. H. Broadbear , C. Duncan , D. Bourton , and S. Rao . 2019. “Avoiding Misdiagnosis When Auditory Verbal Hallucinations Are Present in Borderline Personality Disorder.” Journal of Nervous and Mental Disease 207, no. 12: 1048–1055. 10.1097/NMD.0000000000001073. 31790048

[cpp70051-bib-0007] Beck, A. , R. Steer , and G. Brown . 1996. Manual for the Beck Depression Inventory‐II. Psychological Corporation. 10.1037/t00742-000.

[cpp70051-bib-0008] Beck, A. T. , E. Baruch , J. M. Balter , R. A. Steer , and D. M. Warman . 2004. “A new Instrument for Measuring Insight: The Beck Cognitive Insight Scale.” Schizophrenia Research 68, no. 2–3: 319–329. 10.1016/S0920-9964(03)00189-0. 15099613

[cpp70051-bib-0009] Belohradova Minarikova, K. , J. Prasko , M. Houdkova , et al. 2022. “Hallucinations and Other Psychotic Symptoms in Patients With Borderline Personality Disorder.” Neuropsychiatric Disease and Treatment 18: 787–799. 10.2147/NDT.S360013. 35422622 PMC9005124

[cpp70051-bib-0012] Bohus, M. , N. Kleindienst , M. F. Limberger , et al. 2009. “The Short Version of the Borderline Symptom List (BSL‐23): Development and Initial Data on Psychometric Properties.” Psychopathology 42, no. 1: 32–39. 10.1159/000173701. 19023232

[cpp70051-bib-0014] Can, S. S. , M. İ. Atagün , Ş. A. Korkmaz , and Ç. Soykan . 2019. “Investigating the Jumping to Conclusion Bias in Bipolar Disorder.” Cognitive Neuropsychiatry 24, no. 3: 208–216. 10.1080/13546805.2019.1606708. 30987559

[cpp70051-bib-0015] Catalan, A. , C. J. P. Simons , S. Bustamante , et al. 2015. “Data Gathering Bias: Trait Vulnerability to Psychotic Symptoms?” PLoS ONE 10, no. 7: 1–13. 10.1371/journal.pone.0132442. PMC4493127 26147948

[cpp70051-bib-0016] Cavelti, M. , K. Thompson , C. Hulbert , et al. 2019. “Preliminary Evidence for the Cognitive Model of Auditory Verbal Hallucinations in Youth With Borderline Personality Disorder.” Frontiers in Psychiatry 10: 292. 10.3389/fpsyt.2019.00292. 31156473 PMC6531498

[cpp70051-bib-0017] Cavelti, M. , K. Thompson , C. Hulbert , et al. 2020. “Testing the Interpersonal‐Cognitive Model of Auditory Verbal Hallucinations in Youths With Either Early‐Stage Borderline Personality Disorder or First‐Episode Schizophrenia Spectrum Disorder.” Psychopathology 53, no. 1: 23–35. 10.1159/000505194. 32289803

[cpp70051-bib-0018] Chen, H. , P. Cohen , and S. Chen . 2010. “How Big Is a Big Odds Ratio? Interpreting the Magnitudes of Odds Ratios in Epidemiological Studies.” Communications in Statistics: Simulation and Computation 39, no. 4: 860–864. 10.1080/03610911003650383.

[cpp70051-bib-0019] Colbert, S. M. , and E. R. Peters . 2002. “Need for Closure and Jumping‐To‐Conclusions in Delusion‐Prone Individuals.” Journal of Nervous and Mental Disease 190, no. 1: 27–31. 10.1097/00005053-200201000-00007. 11838027

[cpp70051-bib-0020] D'Aurizio, G. , R. Di Stefano , V. Socci , et al. 2023. “The Role of Emotional Instability in Borderline Personality Disorder: A Systematic Review.” Annals of General Psychiatry 22, no. 1: 9. 10.1186/s12991-023-00439-0. 36918920 PMC10011773

[cpp70051-bib-0021] Diedenhofen, B. , and J. Musch . 2015. “Cocor: A Comprehensive Solution for the Statistical Comparison of Correlations.” PLoS ONE 10, no. 4: e0121945. 10.1371/journal.pone.0121945. 25835001 PMC4383486

[cpp70051-bib-0022] Dudley, R. , P. Taylor , S. Wickham , and P. Hutton . 2016. “Psychosis, Delusions and the ‘Jumping to Conclusions’ Reasoning Bias: A Systematic Review and Meta‐Analysis.” Schizophrenia Bulletin 42, no. 3: 652–665. 10.1093/schbul/sbv150. 26519952 PMC4838082

[cpp70051-bib-0023] Ebner‐Priemer, U. W. , J. Kuo , N. Kleindienst , et al. 2007. “State Affective Instability in Borderline Personality Disorder Assessed by Ambulatory Monitoring.” Psychological Medicine 37, no. 7: 961–970. 10.1017/S0033291706009706. 17202005

[cpp70051-bib-0024] Evans, S. , B. Averbeck , and N. Furl . 2015. “Jumping to Conclusions in Schizophrenia.” Neuropsychiatric Disease and Treatment 11: 1615–1624. 10.2147/ndt.s56870. 26170674 PMC4494619

[cpp70051-bib-0025] Faul, F. , E. Erdfelder , A.‐G. Lang , and A. Buchner . 2007. “G*Power 3: A Flexible Statistical Power Analysis Program for the Social, Behavioral, and Biomedical Sciences.” Behavior Research Methods 39, no. 2: 175–191. 10.3758/BF03193146. 17695343

[cpp70051-bib-0026] Fineberg, S. K. , J. Leavitt , D. S. Stahl , et al. 2018. “Differential Valuation and Learning From Social and Nonsocial Cues in Borderline Personality Disorder.” Biological Psychiatry 84, no. 11: 838–845. 10.1016/j.biopsych.2018.05.020. 30041970 PMC6218635

[cpp70051-bib-0027] First, M. B. , and M. Gibbon . 2004. “The Structured Clinical Interview for DSM‐IV axis I Disorders (SCID‐I) and the Structured Clinical Interview for DSM‐IV axis II Disorders (SCID‐II).” In Comprehensive Handbook of Psychological Assessment, edited by M. J. Hilsenroth and D. L. Segal , vol. 2, 134–143. John Wiley & Sons Inc.

[cpp70051-bib-0028] Garety, P. , T. Ward , R. Emsley , et al. 2021. “Effects of SlowMo, a Blended Digital Therapy Targeting Reasoning, on Paranoia Among People With Psychosis.” JAMA Psychiatry 78, no. 7: 714–725. 10.1001/jamapsychiatry.2021.0326. 33825827 PMC8027943

[cpp70051-bib-0029] Garety, P. A. , D. R. Hemsley , and S. Wessely . 1991. “Reasoning in Deluded Schizophrenic and Paranoid Patients: Biases in Performance on a Probabilistic Inference Task.” Journal of Nervous and Mental Disease 179, no. 4: 194–201. 10.1097/00005053-199104000-00003. 2007889

[cpp70051-bib-0030] Gaweda, T. , K. Prochwicz , M. Krezołek , J. Kłosowska , M. Staszkiewicz , and S. Moritz . 2017. “Self‐Reported Cognitive Distortions in the Psychosis Continuum: A Polish 18‐Item Version of the Davos Assessment of Cognitive Biases Scale (DACOBS‐18).” Schizophrenia Research 192: 317–326. 10.1016/j.schres.2017.05.042. 28601498

[cpp70051-bib-0031] George, A. , and P. H. Soloff . 1986. “Schizotypal Symptoms in Patients With Borderline Personality Disorders.” American Journal of Psychiatry 143, no. 2: 212–215. 10.1176/ajp.143.2.212. 3946657

[cpp70051-bib-0032] Gunderson, J. G. 1979. “The Relatedness of Borderline and Schizophrenic Disorders.” Schizophrenia Bulletin 5, no. 1: 17–22. 10.1093/schbul/5.1.17. 441689

[cpp70051-bib-0033] Gurobi Optimization, LLC 2023 “Gurobi Optimizer Reference Manual” https://www.gurobi.com.

[cpp70051-bib-0112] Hauke, D. J. , V. Roth , P. Karvelis , et al. 2022. “Increased Belief Instability in Psychotic Disorders Predicts Treatment Response to Metacognitive Training.” Schizophrenia Bulletin 48, no. 4: 826–838. 10.1093/schbul/sbac029. 35639557 PMC9212107

[cpp70051-bib-0034] Hayward, M. , A. M. Jones , W. H. Strawson , et al. 2022. “A Cross‐Sectional Study of Auditory Verbal Hallucinations Experienced by People With a Diagnosis of Borderline Personality Disorder.” Clinical Psychology & Psychotherapy 29, no. 2: 631–641. 10.1002/cpp.2655. 34322956

[cpp70051-bib-0035] Henco, L. , A. O. Diaconescu , J. M. Lahnakoski , et al. 2020. “Aberrant Computational Mechanisms of Social Learning and Decision‐Making in Schizophrenia and Borderline Personality Disorder.” PLoS Computational Biology 16, no. 9: 1–22. 10.1371/journal.pcbi.1008162. PMC7588082 32997653

[cpp70051-bib-0036] Henquet, C. , J. van Os , L. K. Pries , et al. 2020. “A Replication Study of JTC bias, Genetic Liability for Psychosis and Delusional Ideation.” Psychological Medicine 52, no. 9: 1777–1783. 33046166 10.1017/S0033291720003578 PMC9280279

[cpp70051-bib-0037] Hepworth, C. R. , K. Ashcroft , and D. Kingdon . 2013. “Auditory Hallucinations: A Comparison of Beliefs About Voices in Individuals With Schizophrenia and Borderline Personality Disorder.” Clinical Psychology & Psychotherapy 20, no. 3: 239–245. 10.1002/cpp.791. 21976361

[cpp70051-bib-0038] Herzog, P. , T. Kube , and E. Fassbinder . 2022. “How Childhood Maltreatment Alters Perception and Cognition ‐ The Predictive Processing Account of Borderline Personality Disorder.” Psychological Medicine 52, no. 14: 2899–2916. 10.1017/S0033291722002458. 35979924 PMC9693729

[cpp70051-bib-0039] Huq, S. F. , P. A. Garety , and D. R. Hemsley . 1988. “Probabilistic Judgements in Deluded and Non‐Deluded Subjects.” Quarterly Journal of Experimental Psychology 40, no. 4: 801–812. 10.1080/14640748808402300. 3212213

[cpp70051-bib-0040] Jacoby, R. J. , J. S. Abramowitz , B. E. Buck , and L. E. Fabricant . 2014. “How Is the Beads Task Related to Intolerance of Uncertainty in Anxiety Disorders?” Journal of Anxiety Disorders 28, no. 6: 495–503. 10.1016/j.janxdis.2014.05.005. 24930046

[cpp70051-bib-0041] Jardri, R. , S. Duverne , A. S. Litvinova , and S. Denève . 2017. “Experimental Evidence for Circular Inference in Schizophrenia.” Nature Communications 8, no. 1: 14218. 10.1038/ncomms14218. PMC5290312 28139642

[cpp70051-bib-0042] Kaiser, D. , G. A. Jacob , G. Domes , and A. Arntz . 2017. “Attentional Bias for Emotional Stimuli in Borderline Personality Disorder: A Meta‐Analysis.” Psychopathology 49, no. 6: 383–396. 10.1159/000448624. PMC5296904 27642753

[cpp70051-bib-0043] Kanske, P. , L. Schulze , I. Dziobek , H. Scheibner , S. Roepke , and T. Singer . 2016. “The Wandering Mind in Borderline Personality Disorder: Instability in Self‐ and Other‐Related Thoughts.” Psychiatry Research 242: 302–310. 10.1016/j.psychres.2016.05.060. 27318635

[cpp70051-bib-0044] Kingdon, D. G. , K. Ashcroft , B. Bhandari , et al. 2010. “Schizophrenia and Borderline Personality Disorder: Similarities and Differences in the Experience of Auditory Hallucinations, Paranoia, and Childhood Trauma.” Journal of Nervous and Mental Disease 198, no. 6: 399–403. 10.1097/NMD.0b013e3181e08c27. 20531117

[cpp70051-bib-0045] Klein, H. S. , and A. E. Pinkham . 2018. “Examining Reasoning Biases in Schizophrenia Using a Modified ‘Jumping to Conclusions’ Probabilistic Reasoning Task.” Psychiatry Research 270: 180–186. 10.1016/j.psychres.2018.09.020. 30261407

[cpp70051-bib-0047] Korn, C. W. , L. La Rosée , H. R. Heekeren , and S. Roepke . 2016. “Social Feedback Processing in Borderline Personality Disorder.” Psychological Medicine 46, no. 3: 575–587. 10.1017/S003329171500207X. 26467724

[cpp70051-bib-0048] Lawrence, K. A. , J. S. Allen , and A. M. Chanen . 2010. “Impulsivity in Borderline Personality Disorder: Reward‐Based Decision‐Making and Its Relationship to Emotional Distress.” Journal of Personality Disorders 24, no. 6: 785–799. 10.1521/pedi.2010.24.6.785. 21158600

[cpp70051-bib-0049] Lazarus, S. A. , J. S. Cheavens , F. Festa , and M. Zachary Rosenthal . 2014. “Interpersonal Functioning in Borderline Personality Disorder: A Systematic Review of Behavioral and Laboratory‐Based Assessments.” Clinical Psychology Review 34, no. 3: 193–205. 10.1016/j.cpr.2014.01.007. 24534643

[cpp70051-bib-0050] Lehrl, S. , G. Triebig , and B. Fischer . 1995. “Multiple Choice Vocabulary Test MWT as a Valid and Short Test to Estimate Premorbid Intelligence.” Acta Neurologica Scandinavica 91, no. 5: 335–345. 10.1111/j.1600-0404.1995.tb07018.x. 7639062

[cpp70051-bib-0052] Liebowitz, M. R. 1979. “Is Borderline a Distinct Entity?” Schizophrenia Bulletin 5, no. 1: 23–38. 10.1093/schbul/5.1.23. 375381

[cpp70051-bib-0053] Linhartová, P. , J. Širůček , A. Ejova , R. Barteček , P. Theiner , and T. Kašpárek . 2021. “Dimensions of Impulsivity in Healthy People, Patients With Borderline Personality Disorder, and Patients With Attention‐Deficit/Hyperactivity Disorder.” Journal of Attention Disorders 25, no. 4: 584–595. 10.1177/1087054718822121. 30628513

[cpp70051-bib-0054] Livet, A. , X. Navarri , S. Potvin , and P. Conrod . 2020. “Cognitive Biases in Individuals With Psychotic‐Like Experiences: A Systematic Review and a Meta‐Analysis.” Schizophrenia Research 222: 10–22. 10.1016/j.schres.2020.06.016. 32595098

[cpp70051-bib-0055] Lunt, L. , J. Bramham , R. G. Morris , et al. 2012. “Prefrontal Cortex Dysfunction and ‘Jumping to Conclusions’: Bias or Deficit?” Journal of Neuropsychology 6, no. 1: 65–78. 10.1111/j.1748-6653.2011.02005.x. 22257612

[cpp70051-bib-0056] McKenna, G. , J. R. E. Fox , and G. Haddock . 2014. “Investigating the ‘Jumping to Conclusions’ Bias in People With Anorexia Nervosa.” European Eating Disorders Review 22, no. 5: 352–359. 10.1002/erv.2310. 25103274

[cpp70051-bib-0057] McLean, B. F. , J. K. Mattiske , and R. P. Balzan . 2017. “Association of the Jumping to Conclusions and Evidence Integration Biases With Delusions in Psychosis: A Detailed Meta‐Analysis.” Schizophrenia Bulletin 43, no. 2: 344–354. 10.1093/schbul/sbw056. 27169465 PMC5605251

[cpp70051-bib-0058] Merrett, Z. , S. L. Rossell , and D. J. Castle . 2016. “Comparing the Experience of Voices in Borderline Personality Disorder With the Experience of Voices in a Psychotic Disorder: A Systematic Review.” Australian &; New Zealand Journal of Psychiatry 50, no. 7: 640–648. 10.1177/0004867416632595. 26912339

[cpp70051-bib-0059] Metzler, P. , and K.‐H. Schmidt . 1992. “Rasch‐Skalierung des Mehrfachwahl‐Wortschatztests (MWT) [Rasch scaling of the Multiple Choice Vocabulary Test].” Diagnostica 38, no. 1: 31–51.

[cpp70051-bib-0060] Moritz, S. , R. P. Balzan , F. Bohn , et al. 2016. “Subjective Versus Objective Cognition: Evidence for Poor Metacognitive Monitoring in Schizophrenia.” Schizophrenia Research 178, no. 1: 74–79. 10.1016/j.schres.2016.08.021. 27591821

[cpp70051-bib-0061] Moritz, S. , A. S. Göritz , R. P. Balzan , Ł. Gawkeda , S. C. Kulagin , and C. Andreou . 2017. “A New Paradigm to Measure Probabilistic Reasoning and a Possible Answer to the Question Why Psychosis‐Prone Individuals Jump to Conclusions.” Journal of Abnormal Psychology 126, no. 4: 406–415. 10.1037/abn0000262. 28277733

[cpp70051-bib-0062] Moritz, S. , G. Pfuhl , T. Lüdtke , M. Menon , R. P. Balzan , and C. Andreou . 2017. “A Two‐Stage Cognitive Theory of the Positive Symptoms of Psychosis. Highlighting the Role of Lowered Decision Thresholds.” Journal of Behavior Therapy and Experimental Psychiatry 56: 12–20. 10.1016/j.jbtep.2016.07.004. 27501907

[cpp70051-bib-0063] Moritz, S. , F. Scheu , C. Andreou , U. Pfueller , M. Weisbrod , and D. Roesch‐Ely . 2016. “Reasoning in Psychosis: Risky but Not Necessarily Hasty.” Cognitive Neuropsychiatry 21, no. 2: 91–106. 10.1080/13546805.2015.1136611. 26884221

[cpp70051-bib-0064] Moritz, S. , J. Scheunemann , T. Lüdtke , et al. 2020. “Prolonged Rather Than Hasty Decision‐Making in Schizophrenia Using the Box Task. Must We Rethink the Jumping to Conclusions Account of Paranoia?” Schizophrenia Research 222: 202–208. 10.1016/j.schres.2020.05.056. 32507550

[cpp70051-bib-0065] Moritz, S. , L. Schilling , K. Wingenfeld , et al. 2011. “Psychotic‐Like Cognitive Biases in Borderline Personality Disorder.” Journal of Behavior Therapy and Experimental Psychiatry 42, no. 3: 349–354. 10.1016/j.jbtep.2011.02.003. 21411041

[cpp70051-bib-0066] Moritz, S. , N. Van Quaquebeke , and T. M. Lincoln . 2012. “Jumping to Conclusions Is Associated With Paranoia but Not General Suspiciousness: A Comparison of Two Versions of the Probabilistic Reasoning Paradigm.” Schizophrenia Research and Treatment 2012: 384039. 10.1155/2012/384039. 23125930 PMC3483676

[cpp70051-bib-0067] Moritz, S. , and T. S. Woodward . 2005. “Jumping to Conclusions in Delusional and non‐delusional Schizophrenic Patients.” British Journal of Clinical Psychology 44, no. 2: 193–207. 10.1348/014466505X35678. 16004654

[cpp70051-bib-0068] Moritz, S. , and T. S. Woodward . 2007. “Metacognitive Training in Schizophrenia: From Basic Research to Knowledge Translation and Intervention.” Current Opinion in Psychiatry 20, no. 6: 619–625. 10.1097/YCO.0b013e3282f0b8ed. 17921766

[cpp70051-bib-0069] Moritz, S. , T. S. Woodward , and D. Hausmann . 2006. “Incautious Reasoning as a Pathogenetic Factor for the Development of Psychotic Symptoms in Schizophrenia.” Schizophrenia Bulletin 32, no. 2: 327–331. 10.1093/schbul/sbj034. 16339971 PMC2632219

[cpp70051-bib-0071] Niemantsverdriet, M. B. A. , C. W. Slotema , J. D. Blom , et al. 2017. “Hallucinations in Borderline Personality Disorder: Prevalence, Characteristics and Associations With Comorbid Symptoms and Disorders.” Scientific Reports 7, no. 1: 13920. 10.1038/s41598-017-13108-6. 29066713 PMC5654997

[cpp70051-bib-0072] Patton, J. H. , M. S. Stanford , and E. S. Barratt . 1995. “Factor Structure of the Barratt Impulsiveness Scale.” Journal of Clinical Psychology 51, no. 6: 768–774. 10.1002/1097-4679(199511)51:6<768::AID-JCLP2270510607>3.0.CO;2-1. 8778124

[cpp70051-bib-0073] Peters, E. R. , S. Moritz , M. Schwannauer , et al. 2014. “Cognitive Biases Questionnaire for Psychosis.” Schizophrenia Bulletin 40, no. 2: 300–313. 10.1093/schbul/sbs199. 23413104 PMC3932080

[cpp70051-bib-0074] Pulcu, E. , K. E. A. Saunders , C. J. Harmer , et al. 2022. “Using a Generative Model of Affect to Characterize Affective Variability and Its Response to Treatment in Bipolar Disorder.” Proceedings of the National Academy of Sciences of the United States of America 119, no. 28: e2202983119. 10.1073/pnas.2202983119. 35787043 PMC9282445

[cpp70051-bib-0075] Puri, P. , D. Kumar , K. Muralidharan , and M. T. Kishore . 2018. “Individuals With Borderline Personality Disorder Manifest Cognitive Biases Implicated in Psychosis.” Psychiatry Research 267: 414–419. 10.1016/j.psychres.2018.06.040. 29960939

[cpp70051-bib-0076] Puri, P. , D. Kumar , K. Muralidharan , and M. T. Kishore . 2021. “Evaluating Schema Modes and Cognitive Distortions in Borderline Personality Disorder: A Mixed‐Method Approach.” Journal of Clinical Psychology 77, no. 9: 1973–1984. 10.1002/jclp.23126. 33625735

[cpp70051-bib-0077] Pytlik, N. , D. Soll , K. Hesse , et al. 2020. “Problems in Measuring the JTC‐Bias in Patients With Psychotic Disorders With the Fish Task: A Secondary Analysis of a Baseline Assessment of a Randomized Controlled Trial.” BMC Psychiatry 20, no. 1: 554. 10.1186/s12888-020-02959-5. 33228583 PMC7685639

[cpp70051-bib-0078] Randolph, J. J. , K. Falbe , A. K. Manuel , and J. L. Balloun . 2014. “A Step‐By‐Step Guide to Propensity Score Matching in R.” Practical Assessment, Research & Evaluation 19: 18. 10.7275/n3pv-tx27.

[cpp70051-bib-0079] Reitan, R. M. , and D. Wolfson . 1995. “Category Test and Trail Making Test as Measures of Frontal Lobe Functions.” Clinical Neuropsychologist 9, no. 1: 50–56. 10.1080/13854049508402057.

[cpp70051-bib-0081] Renneberg, B. , and A. Seehausen . 2010. “Fragebogen zu Gedanken und Gefühlen (FGG) [Questionnaire of Thoughts and Feelings (QTF)—A screening instrument for borderline‐specific cognitions].” Zeitschrift für Klinische Psychologie und Psychotherapie 39, no. 3: 170–178. 10.1026/1616-3443/a000031.

[cpp70051-bib-0083] Scheunemann, J. , Ł. Gawęda , K. M. Reininger , L. Jelinek , H. Hildebrandt , and S. Moritz . 2020. “Advice Weighting as a Novel Measure for Belief Flexibility in People With Psychotic‐Like Experiences.” Schizophrenia Research 216: 129–137. 10.1016/j.schres.2019.12.016. 31924370

[cpp70051-bib-0113] Scheunemann, J. , L. Jelinek , S. V. Biedermann , et al. 2023. “Can You Trust This Source? Advice Taking in Borderline Personality Disorder.” European Archives of Psychiatry and Clinical Neuroscience 273, no. 4: 875–885. 10.1007/s00406-022-01539-w. 36629942 PMC10238350

[cpp70051-bib-0084] Schilling, L. , S. Moritz , L. Kriston , M. Krieger , and M. Nagel . 2018. “Efficacy of Metacognitive Training for Patients With Borderline Personality Disorder: Preliminary Results.” Psychiatry Research 262: 459–464. 10.1016/j.psychres.2017.09.024. 28927866

[cpp70051-bib-0085] Schilling, L. , S. Moritz , B. Schneider , J. Bierbrodt , and M. Nagel . 2015. “Attributional ‘Tunnel Vision’ in Patients With Borderline Personality Disorder.” Journal of Personality Disorders 29, no. 6: 839–846. 10.1521/pedi_2015_29_181. 25710733

[cpp70051-bib-0086] Serrano‐Guerrero, E. , M. Ruiz‐Veguilla , A. Martín‐Rodríguez , and J. F. Rodríguez‐Testal . 2020. “Inflexibility of Beliefs and Jumping to Conclusions in Active Schizophrenia.” Psychiatry Research 284: 112776. 10.1016/j.psychres.2020.112776. 31981941

[cpp70051-bib-0115] Sheehan, D. V. , Y. Lecrubier , K. H. Sheehan , et al. 1998. “The Mini‐International Neuropsychiatric Interview (M.I.N.I.): The Development and Validation of a Structured Diagnostic Psychiatric Interview for DSM‐IV and ICD‐10.” Journal of clinical psychiatry 59, no. Suppl 20: 22–57. 9881538

[cpp70051-bib-0087] Slotema, C. W. , H. Bayrak , M. M. J. Linszen , M. Deen , and I. E. C. Sommer . 2019. “Hallucinations in Patients With Borderline Personality Disorder: Characteristics, Severity, and Relationship With Schizotypy and Loneliness.” Acta Psychiatrica Scandinavica 139, no. 5: 434–442. 10.1111/acps.13012. 30712290

[cpp70051-bib-0088] Slotema, C. W. , K. Daalman , J. D. Blom , K. M. Diederen , H. W. Hoek , and I. E. C. Sommer . 2012. “Auditory Verbal Hallucinations in Patients With Borderline Personality Disorder Are Similar to Those in Schizophrenia.” Psychological Medicine 42, no. 9: 1873–1878. 10.1017/S0033291712000165. 22336487

[cpp70051-bib-0090] So, S. H. , N. Y. Siu , H. Wong , W. Chan , and P. A. Garety . 2016. “‘Jumping to Conclusions’ Data‐Gathering Bias in Psychosis and Other Psychiatric Disorders — Two Meta‐Analyses of Comparisons Between Patients and Healthy Individuals.” Clinical Psychology Review 46: 151–167. 10.1016/j.cpr.2016.05.001. 27216559

[cpp70051-bib-0091] Speechley, W. J. , J. C. Whitman , and T. S. Woodward . 2010. “The Contribution of Hypersalience to the ‘Jumping to Conclusions’ Bias Associated With Delusions in Schizophrenia.” Journal of Psychiatry & Neuroscience 35, no. 1: 7–17. 10.1503/jpn.090025. 20040242 PMC2799500

[cpp70051-bib-0095] Strawson, W. H. , H.‐T. Wang , L. Quadt , et al. 2022. “Voice Hearing in Borderline Personality Disorder Across Perceptual, Subjective, and Neural Dimensions.” International Journal of Neuropsychopharmacology 25, no. 5: 375–386. 10.1093/ijnp/pyab093. 34940826 PMC9154289

[cpp70051-bib-0096] Svaldi, J. , A. Philipsen , and S. Matthies . 2012. “Risky Decision‐Making in Borderline Personality Disorder.” Psychiatry Research 197, no. 1–2: 112–118. 10.1016/j.psychres.2012.01.014. 22421066

[cpp70051-bib-0098] Tripoli, G. , D. Quattrone , G. Murray , et al. 2019. “Jumping to Conclusions, General Intelligence, and Psychosis Liability: Findings From the Multi‐Centre EU‐GEI Case‐Control Study.” Psychological Medicine 51, no. 4: 623–633. 10.1093/schbul/sbz019.322. PMC8020493 32327005

[cpp70051-bib-0099] Tschoeke, S. , T. Steinert , E. Flammer , and C. Uhlmann . 2014. “Similarities and Differences in Borderline Personality Disorder and Schizophrenia With Voice Hearing.” Journal of Nervous and Mental Disease 202, no. 7: 544–549. 10.1097/NMD.0000000000000159. 24921419

[cpp70051-bib-0100] Van Dael, F. , D. Versmissen , I. Janssen , I. Myin‐Germeys , J. Van Os , and L. Krabbendam . 2006. “Data Gathering: Biased in Psychosis?” Schizophrenia Bulletin 32, no. 2: 341–351. 10.1093/schbul/sbj021. 16254066 PMC2632225

[cpp70051-bib-0102] Veckenstedt, R. , S. Randjbar , F. Vitzthum , B. Hottenrott , T. S. Woodward , and S. Moritz . 2011. “Incorrigibility, Jumping to Conclusions, and Decision Threshold in Schizophrenia.” Cognitive Neuropsychiatry 16, no. 2: 174–192. 10.1080/13546805.2010.536084. 21271413

[cpp70051-bib-0103] Waller, N. G. , F. W. Putnam , and E. B. Carlson . 1996. “Types of Dissociation and Dissociative Types: A Taxometric Analysis of Dissociative Experiences.” Psychological Methods 1, no. 3: 300–321. 10.1037/1082-989X.1.3.300.

[cpp70051-bib-0104] Ward, T. , and P. A. Garety . 2019. “Fast and Slow Thinking in Distressing Delusions: A Review of the Literature and Implications for Targeted Therapy.” Schizophrenia Research 203: 80–87. 10.1016/j.schres.2017.08.045. 28927863 PMC6336980

[cpp70051-bib-0105] Warman, D. M. , and J. M. Martin . 2006. “Jumping to Conclusions and Delusion Proneness: The Impact of Emotionally Salient Stimuli.” Journal of Nervous and Mental Disease 194, no. 10: 760–765. 10.1097/01.nmd.0000239907.83668.aa. 17041288

[cpp70051-bib-0106] Wechsler, D. 1981. “The Psychometric Tradition: Developing the Wechsler Adult Intelligence Scale.” Contemporary Educational Psychology 6, no. 2: 82–85. 10.1016/0361-476X(81)90035-7.

[cpp70051-bib-0107] Westermann, S. , S. Salzmann , X. Fuchs , and T. M. Lincoln . 2012. “Introducing a Social Beads Task.” Journal of Experimental Psychopathology 3, no. 4: 594–611. 10.5127/jep.017111.

[cpp70051-bib-0108] Wilson, B. , J. Cockburn , A. Baddeley , and R. Hiorns . 1989. “The Development and Validation of a Test Battery for Detecting and Monitoring Everyday Memory Problems.” Journal of Clinical and Experimental Neuropsychology 11, no. 6: 855–870. 10.1080/01688638908400940. 2592527

[cpp70051-bib-0109] Woodward, T. S. , M. Munz , C. LeClerc , and T. Lecomte . 2009. “Change in Delusions is Associated With Change in ‘Jumping to Conclusions’.” Psychiatry Research 170, no. 2–3: 124–127. 10.1016/j.psychres.2008.10.020. 19906443

[cpp70051-bib-0110] Zanarini, M. C. , J. G. Gunderson , and F. R. Frankenburg . 1990. “Cognitive Features of Borderline Personality Disorder.” American Journal of Psychiatry 147, no. 1: 57–63. 10.1176/ajp.147.1.57. 2293789

[cpp70051-bib-0111] Zandersen, M. , M. G. Henriksen , and J. Parnas . 2019. “A Recurrent Question: What Is Borderline?” Journal of Personality Disorders 33, no. 3: 341–369. 10.1521/pedi_2018_32_348. 29469662

