# Supplementary material for: Psychotic‐Like Reasoning Styles in Patients With Borderline Personality Disorder? An Experimental Investigation of the Jumping to Conclusions Bias
Source: Clin Psychol Psychother. 2025 Mar 5;32(2):e70051. doi: 10.1002/cpp.70051 (PMC11881219; doi:10.1002/cpp.70051)
Supplement: Supplementary file 1 — Data S1. Psychopathological and neuropsychological measures. [file CPP-32-e70051-s002.pdf]

Supplementary Material 1. Psychopathological and neuropsychological measures.

1.1 Borderline Symptom List (BSL-23)

The Borderline Symptom List (BSL-23; Bohus et al., 2009) is the abbreviated version of the Borderline Symptom List-95 (Bohus et al., 2001) and consists of 23 items. On a five-point Likert scale ranging from 0 (not at all) to 4 (very much), patients indicate how much they have suffered from various symptoms characteristic of BPD over the past seven days. The BSL-23 shows good criterion and construct validity. The internal consistency was between $\alpha = 0.94$ and $\alpha = 0.97$ in the validation study (Bohus et al., 2009) and $\alpha = 0.93$ in the current patient sample. A total of 160 patients completed the BSL-23.

1.2 Beck Depression Inventory-II (BDI-II)

The Beck Depression Inventory II (BDI-II; Beck et al., 1996) is a questionnaire that assesses the severity of depressive symptoms. The BDI-II contains of 21 items that can be allocated to the 9 symptoms of depression according to the DSM-IV criteria. Items are scored from 0 to 3, with higher values indicating higher symptomology. The BDI-II shows good criterion and construct validity (Storch et al., 2004). The internal consistency was $\alpha = 0.90$ in a validation study (Storch et al., 2004) and $\alpha = 0.85$ in the current patient sample. A total of 156 patients completed the BDI-II.

1.3 Dissociative Experiences Scale-Taxon (DES-T)

The Dissociative Experiences Scale-Taxon (DES-T; Waller et al., 1996) consists of eight items representing indicators of pathological dissociation extracted from the 28-item Dissociative Experiences Scale (Bernstein & Putnam, 1986). In a 2015 study in Germany, the DES-T was psychometrically evaluated in a large general population sample ($n = 2359$; Spitzer et al., 2015). The study showed good internal consistency (Cronbach's $\alpha = 0.92$), with discriminant coefficients exceeding 0.65. The internal consistency was $\alpha = 0.80$ in our current sample, with a total of 151 patients completing the DES-T.

1.4 Barratt Impulsiveness Scale (BIS)

The Barratt Impulsiveness Scale (BIS; Barratt, 1959) is a 30-item self-report questionnaire designed to measure impulsivity. On a 4-point Likert scale ranging from 1 (rarely/never) to 4 (almost always/always), participants indicate how often they engage in impulsive behavior. We used the total score of the 11th revision (Patton et al., 1995). The BIS shows good criterion and construct validity (for a review, see Vasconcelos et al., 2012). The internal consistency was α = 0.83 in a large replication study (Stanford et al., 2009) and α = 0.76 in the current sample comprised of 46 patients.

1.5 Questionnaire of Thoughts and Feelings (QTF)

We used the 14-item short version of the Questionnaire of Thoughts and Feelings (QTF; Renneberg & Seehausen, 2010; for the original 34-item version, see Renneberg et al. [2005]) to capture borderline-specific cognitions. Participants rate statements on a 5-point Likert-scale from 1 (I do not agree at all) to 5 (I agree completely), with higher scores indicating more pronounced borderline-specific cognitions. In the validation study, the QTF shows good criterion and construct validity. The internal consistency was high, both in the validation study (α = 0.96; Renneberg & Seehausen, 2010) and in the patient sample of the current study (α = 0.86). We have data from 64 patients on the QTF.

1.6 Beck Cognitive Insight Scale (BCIS)

With the goal of measuring insight in patients with psychotic experiences, the Beck Cognitive Insight Scale (BCIS; Beck et al., 2004) measures, on two subscales, the patients' self-reflectiveness (9 items) and self-certainty (6 items) in their interpretations of their experiences. The questionnaire uses a 4-point Likert scale from 0 (do not agree at all) to 3 (agree completely). High scores on the subscale self-reflectiveness indicate good insight, whereas high scores on the subscale self-certainty indicate low insight. The BCIS shows good criterion and construct validity (Riggs et al., 2010). The internal consistency for self-reflectiveness was α = 0.68 in the validation study (Beck et al., 2004) and α = 0.42 in the current sample; for self-certainty, the internal consistency was α = 0.60 in the validation study

(Beck et al., 2004) and α = 0.51 in the current sample. A total of 71 patients completed the BCIS.

1.7 Cognitive Biases Questionnaire for psychosis (CBQp)

The Cognitive Biases Questionnaire for psychosis (CBQp; Peters et al., 2014) was developed to assess five cognitive biases (jumping to conclusions, intentionalizing, catastrophizing, emotional reasoning, and dichotomous thinking) relevant to the development of psychosis (Peters et al., 2010). The CBQp resembles the Cognitive Style Test (Blackburn et al., 1986). Participants read 30 vignettes and choose one of three statements illustrating the absence of bias (scored as 1), the possible presence of bias (scored as 2), and the likely presence of bias (scored as 3). The CBQp shows good criterion and construct validity (Peters et al., 2014). The internal consistency was α = 0.89 in the validation study (Peters et al., 2014) and α = 0.87 in the current patient sample. A total of 28 patients completed the CBQp.

1.8 Vocabulary tests as IQ estimates (MWT-B and WST)

As a measure of premorbid intelligence, we used two German multiple-choice vocabulary tests. For each item, participants had to identify which word out of five words actually exists (the other four words are fictional neologisms). The Wortschatztest (WST; Metzler & Schmidt, 1992) consists of 42 items, and 62 participants completed the test. The Mehrfachwahl-Wortschatz-Intelligenztest (MWT-B; Lehrl et al., 1995) is 37 items long, and 109 participants completed it. For both tests, we report raw values.

1.9 Rivermead Behavioural Memory Test (RBMT)

The Rivermead Behavioural Memory Test (RBMT; Wilson et al., 1989) assesses everyday memory using tasks that mimic everyday challenges. The RBMT is a short memory battery consisting of 12 tests in which the patient must remember a first name and a last name, remember a short newspaper article, remember a route, recognize objects and faces, remember to ask for a hidden object, ask a question when an alarm rings, and answer

questions about time and space orientation. Because it simulates everyday tasks, the RBMT is considered an ecological memory battery with high face validity (Koltai et al., 1996), and it has good psychometric properties (Wilson et al., 1989). We report the raw data of n = 142 patients.

1.10 Trail Making Test (TMT)

The Trail Making Test (TMT; Reitan & Wolfson, 1995) consists of two subtests (TMT-A and TMT-B). For the TMT-A, the participant uses a pen to connect 25 randomly arranged numbers in ascending order on a standard-sized paper (DIN A 4) as a measure of processing speed. For the TMT-B, the participant connects 13 numbers with 13 letters alternately in ascending order (A-1-B-2-. . .-M-13) to measure execute functioning. We report both the raw time and the age-adjusted percentile for both tests (norm values are derived from a sample of n = 911; Tombaugh, 2004) with n = 168 for TMT-A and n = 169 for TMT-B.

1.11 Digits forward (DF) and Digits backward (DB)

The digits forward (DF) digit backward (DB) are tests designed to assess attention and working memory and are taken from the Wechsler Adult Intelligence Scale-Revised (WAIS-R; Wechsler, 1981). In the first part, the participant is asked to repeat a series of randomly arranged digits "forward" immediately following a single reading by the examiner (DF). The DB follows the same principle, with the difference that the respondent repeats the sequence in reverse order, starting with the last number. We used the raw scores (number of correctly remembered items) and had data from 81 patients in total.

References

Barratt, E. S. (1959). Anxiety and Impulsiveness Related to Psychomotor Efficiency. *Perceptual and Motor Skills*, *9*(3). https://doi.org/10.2466/pms.1959.9.3.191

Beck, A., Steer, R., & Brown, G. (1996). *Manual for the Beck depression inventory-II*. Psychological Corporation. https://doi.org/10.1037/t00742-000

Beck, A. T., Baruch, E., Balter, J. M., Steer, R. A., & Warman, D. M. (2004). A new instrument for measuring insight: The Beck Cognitive Insight Scale. *Schizophrenia Research*, *68*(2–3), 319–329. https://doi.org/10.1016/S0920-9964(03)00189-0

Bernstein, E. M., & Putnam, F. W. (1986). Development, reliability, and validity of a dissociation scale. *Journal of Nervous and Mental Disease*, *174*(12). https://doi.org/10.1097/00005053-198612000-00004

Blackburn, I. M., Jones, S., & Lewin, R. J. P. (1986). Cognitive style in depression. *British Journal of Clinical Psychology*, *25*(4). https://doi.org/10.1111/j.2044-8260.1986.tb00704.x

Bohus, M., Kleindienst, N., Limberger, M. F., Stieglitz, R.-D., Domsalla, M., Chapman, A. L., Steil, R., Philipsen, A., & Wolf, M. (2009). The Short Version of the Borderline Symptom List (BSL-23): Development and Initial Data on Psychometric Properties. *Psychopathology*, *42*(1), 32–39. https://doi.org/10.1159/000173701

Bohus, M., Limberger, M. F., Frank, U., Sender, I., Gratwohl, T., & Stieglitz, R. D. (2001). Entwicklung der borderline-symptom-liste. *PPmP Psychotherapie Psychosomatik Medizinische Psychologie*, *51*(5). https://doi.org/10.1055/s-2001-13281

Koltai, D. C., Bowler, R. M., & Shore, M. D. (1996). The Rivermead behavioural memory test and Wechsler memory scale-revised: Relationship to everyday memory impairment. *Assessment*, *3*(4). https://doi.org/10.1177/107319119600300410

Lehrl, S., Triebig, G., & Fischer, B. (1995). Multiple choice vocabulary test MWT as a valid

and short test to estimate premorbid intelligence. *Acta Neurologica Scandinavica*, *91*(5). https://doi.org/10.1111/j.1600-0404.1995.tb07018.x

Metzler, P., & Schmidt, K.-H. (1992). Rasch-Skalierung des Mehrfachwahl-Wortschatztests (MWT). *Diagnostica*, *38*(1).

Patton, J. H., Stanford, M. S., & Barratt, E. S. (1995). Factor structure of the barratt impulsiveness scale. *Journal of Clinical Psychology*, *51*(6). https://doi.org/10.1002/1097-4679(199511)51:6<768::AID-JCLP2270510607>3.0.CO;2-1

Peters, E. R., Moritz, S., Schwannauer, M., Wiseman, Z., Greenwood, K. E., Scott, J., Beck, A. T., Donaldson, C., Hagen, R., Ross, K., Veckenstedt, R., Ison, R., Williams, S., Kuipers, E., & Garety, P. A. (2014). Cognitive biases questionnaire for psychosis. *Schizophrenia Bulletin*, *40*(2). https://doi.org/10.1093/schbul/sbs199

Reitan, R. M., & Wolfson, D. (1995). Category Test and Trail Making Test as Measures of Frontal Lobe Functions. *The Clinical Neuropsychologist*, *9*(1). https://doi.org/10.1080/13854049508402057

Renneberg, B., Schmidt-Rathjens, C., Hippin, R., Backenstrass, M., & Fydrich, T. (2005). Cognitive characteristics of patients with borderline personality disorder: Development and validation of a self-report inventory. *Journal of Behavior Therapy and Experimental Psychiatry*, *36*(3). https://doi.org/10.1016/j.jbtep.2005.05.001

Renneberg, B., & Seehausen, A. (2010). Fragebogen zu Gedanken und Gefühlen (FGG). *Zeitschrift Für Klinische Psychologie Und Psychotherapie*, *39*(3). https://doi.org/10.1026/1616-3443/a000031

Riggs, S. E., Grant, P. M., Perivoliotis, D., & Beck, A. T. (2010). Assessment of cognitive insight: a qualitative review. *Schizophrenia Bulletin*, *38*(2), 338–350.

Spitzer, C., Freyberger, H., Brähler, E., Beutel, M. E., & Stieglitz, R. (2015). Psychometric Evaluation of the Dissociative Experiences Scale-Taxon (DES-T). *PPmP Psychotherapie Psychosomatik Medizinische Psychologie*, *65*(3–4).

https://doi.org/10.1055/s-0034-1395690

Stanford, M. S., Mathias, C. W., Dougherty, D. M., Lake, S. L., Anderson, N. E., & Patton, J. H. (2009). Fifty years of the Barratt Impulsiveness Scale: An update and review. In *Personality and Individual Differences* (Vol. 47, Issue 5). https://doi.org/10.1016/j.paid.2009.04.008

Storch, E. A., Roberti, J. W., & Roth, D. A. (2004). Factor structure, concurrent validity, and internal consistency of the Beck Depression Inventory - Second Edition in a sample of college students. *Depression and Anxiety*, *19*(3). https://doi.org/10.1002/da.20002

Tombaugh, T. N. (2004). Trail Making Test A and B: Normative data stratified by age and education. *Archives of Clinical Neuropsychology*, *19*(2). https://doi.org/10.1016/S0887-6177(03)00039-8

Vasconcelos, A. G., Malloy-Diniz, L., & Correa, H. (2012). Systematic review of psychometric proprieties of barrattimpulsiveness scale version 11 (BIS-11). *Clinical Neuropsychiatry*, *9*(2).

Waller, N. G., Putnam, F. W., & Carlson, E. B. (1996). Types of dissociation and dissociative types: A taxometric analysis of dissociative experiences. *Psychological Methods*, *1*(3). https://doi.org/10.1037/1082-989X.1.3.300

Wechsler, D. (1981). The psychometric tradition: Developing the wechsler adult intelligence scale. In *Contemporary Educational Psychology* (Vol. 6, Issue 2). https://doi.org/10.1016/0361-476X(81)90035-7

Wilson, B., Cockburn, J., Baddeley, A., & Hiorns, R. (1989). The development and validation of a test battery for detecting and monitoring everyday memory problems. *Journal of Clinical and Experimental Neuropsychology*, *11*(6). https://doi.org/10.1080/01688638908400940
