# Supplementary material for: Psychotic‐Like Reasoning Styles in Patients With Borderline Personality Disorder? An Experimental Investigation of the Jumping to Conclusions Bias
Source: Clin Psychol Psychother. 2025 Mar 5;32(2):e70051. doi: 10.1002/cpp.70051 (PMC11881219; doi:10.1002/cpp.70051)
Supplement: Supplementary file 2 — Table S1. Frequency of comorbidities based on the Mini International Neuropsychiatric Interview (M.I.N.I.) in a sub sample of n = 159 patients (93.5% of total BPD sample). [file CPP-32-e70051-s001.pdf]

Supplementary Material 2. Frequency of comorbidities based on the Mini International Neuropsychiatric Interview (M.I.N.I.).

Table S1

Frequency of comorbidities based on the Mini International Neuropsychiatric Interview (M.I.N.I.) in a subsample of n = 159 patients (93.5% of total BPD sample).

Any comorbidity	*n* = 154	96.9%
Any depressive disorder (lifetime)	*n* = 147	92.5%
Current depression	*n* = 120	75.5%
Lifetime manic or hypomanic episode	*n* = 14	8.8%
Posttraumatic stress disorder	*n* = 24	15.1%
Any anxiety disorder	*n* = 14	8.8%
Obsessive-compulsive disorder	*n* = 2	1.3%
Any alcohol-use disorder	*n* = 41	25.8%
Any substance-use disorder (non-alcohol)	*n* = 29	18.2%
Any eating disorder	*n* = 3	1.9%
Mean number of comorbidities[a] in addition to BPD diagnosis	*M* = 2.5	*SD* = 1.2

Note. [a] as categorized above.
